# Supplementary material for: Approximated gene expression trajectories for gene regulatory network inference on cell tracks
Source: iScience. 2024 Aug 30;27(9):110840. doi: 10.1016/j.isci.2024.110840 (PMC11407030; doi:10.1016/j.isci.2024.110840)
Supplement: Document S1. Figures S1–S8 and Table S1 [file mmc1.pdf]

iScience, Volume *27*

Supplemental information

Approximated gene expression trajectories for gene regulatory network inference on cell tracks

Kay Spiess, Shannon E. Taylor, Timothy Fulton, Kane Toh, Dillan Saunders, Seongwon Hwang, Yuxuan Wang, Brooks Paige, Benjamin Steventon, and Berta Verd

Approximated Gene Expression Trajectories (AGETs) for Gene Regulatory Network Inference on Cell Tracks

Kay Spiess[1,2#], Shannon E. Taylor[3#], Timothy Fulton[1], Kane Toh[1], Dillan Saunders[1], Seongwon Hwang[1], Yuxuan Wang[1], Brooks Paige[2,4*], Benjamin Steventon[1*] and Berta Verd[1,3*]

[1]Department of Genetics, University of Cambridge, Cambridge, UK
[2]The Alan Turing Institute, London, UK;
[3]Department of Biology, University of Oxford, UK;
[4]Centre for Artificial Intelligence, University College London, UK

[*]To whom correspondence should be addressed:
berta.verdfernandez@biology.ox.ac.uk
[#]Equal contribution

Supplementary Information

		tbxta	tbx16	tbx6
Inter-connectivity matrix W	tbxta	0.665	0.305	1.52
	tbx16	-2.27	0.00816	-12.3
	tbx6	-10.9	-0.796	0.00110
External Input E	Wnt	1.66	0.511	6.63
	FGF	0.815	0.791	8.76
Production rate	R	28.2	160	2.73
Degradation rate	λ	5.15	87.4	1.66
Basal production	h	0.0612	-0.923	-0.899

Table 1: **Parameter values of the MAP network.** Related to Figure 3.

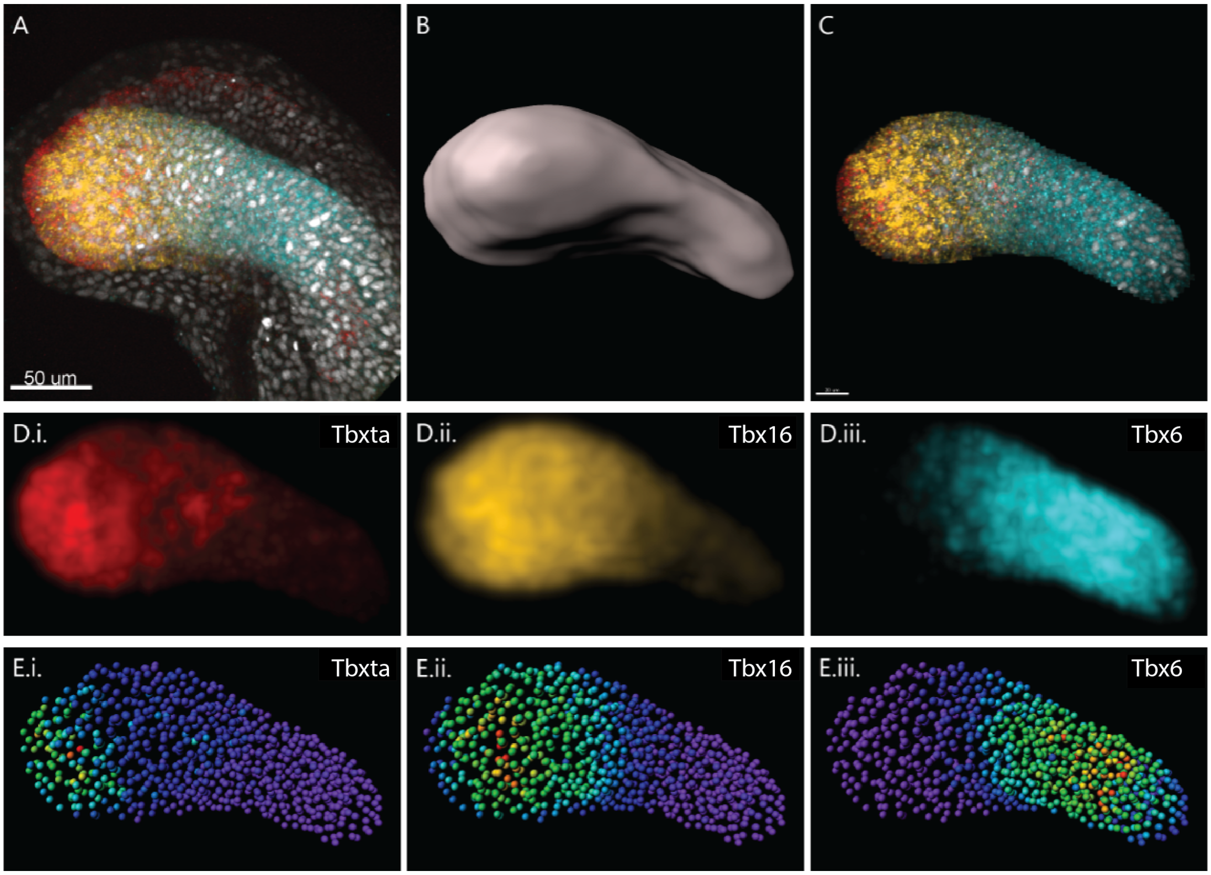

Figure S 1: **Gene expression data preparation pipeline.** Related to Figure 2. **A.** Typical HCR image of a 22 somite stage zebrafish embryo tailbud stained for *tbxta* (red), *tbx16* (yellow), *tbx6* (blue) and DAPI (gray). Anterior to the right, posterior to the left, dorsal up and ventral down from here on. **B.** Surface masking the PSM based on T-box gene expression and morphological landmarks. **C.** Gene expression and nuclear marker in the masked PSM (as before *tbxta* in red, *tbx16* in yellow, *tbx6* in blue and DAPI in gray). **D.** Normalising gene expression levels: *tbxta* and *tbx16* levels in the anterior PSM are normalised to zero while posterior PSM levels of *tbx6* are normalised to zero, to eliminate background expression. A Gaussian filter has been then applied to each T-box gene to smoothen gene expression across the PSM. **E.** Nuclei are segmented using the DAPI channel creating spots in 3D space. Spots are coloured according to the median intensity of each gene i. *tbxta*, ii. *tbx16* and iii. *tbx6*), where purple denotes zero expression and red 1, which is the highest expression. The spatial coordinates of the spots together with the median intensities were exported and used to generate the AGETs.

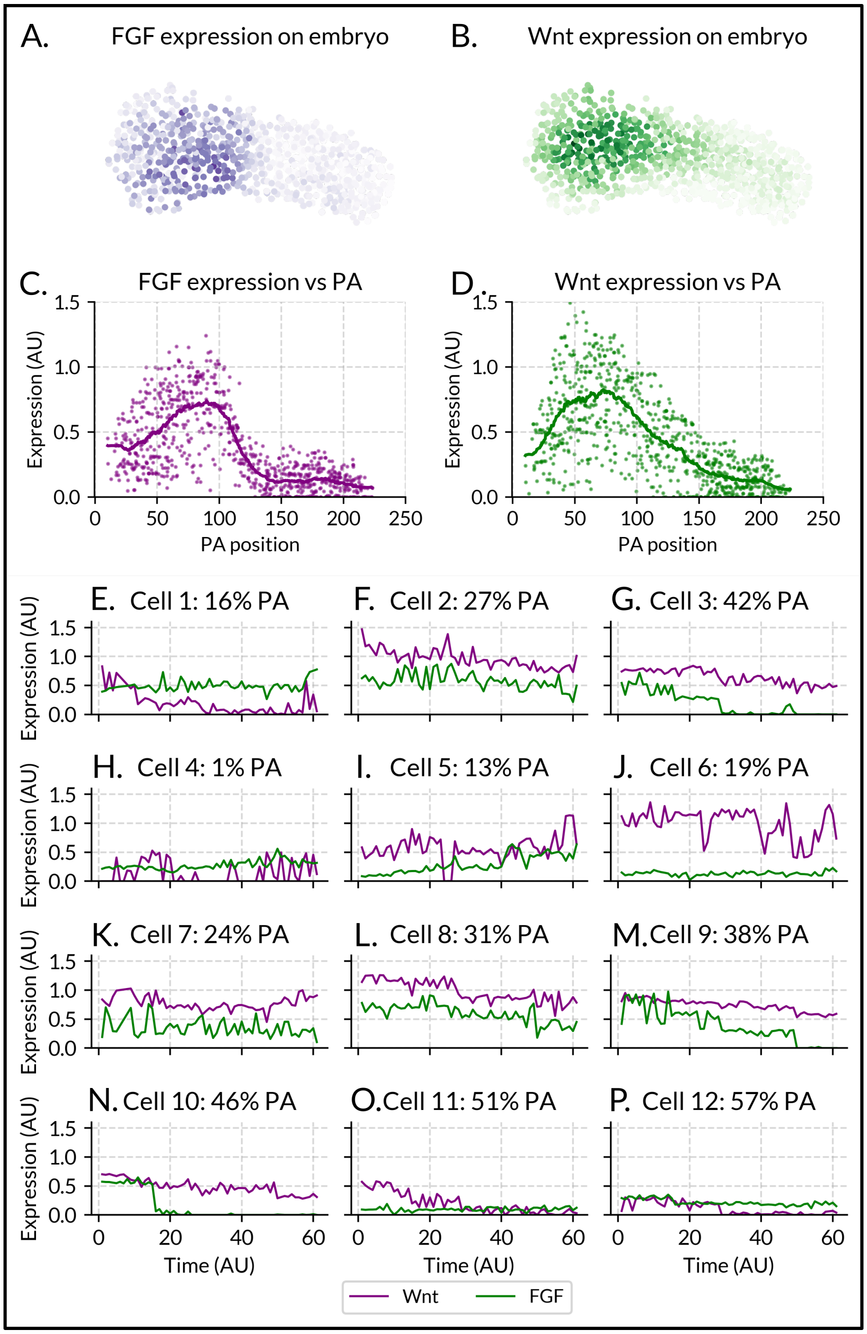

Figure S 2: **Signalling AGETs.** Related to Figure 3. **A.** Approximated FGF (purple) and **B.** Wnt (green) expression at the final time point of the AGETs, plotted onto cell position (X and Y axes). The intensity of the colour of each point represents the approximated signalling value in that cell (between 0 and 1.5 AU.) **C.** Quantification of FGF and **D.** Wnt expression along the posterio-to-anterior axis of the PSM at the final time point of the signalling AGETs. Solid line shows the rolling average gene expression across the PA axis, and individual dots represent the PA position of individual cells. **E.** FGF and **F.** Wnt expression in the individual AGETs for the cells shown in Figure 3.

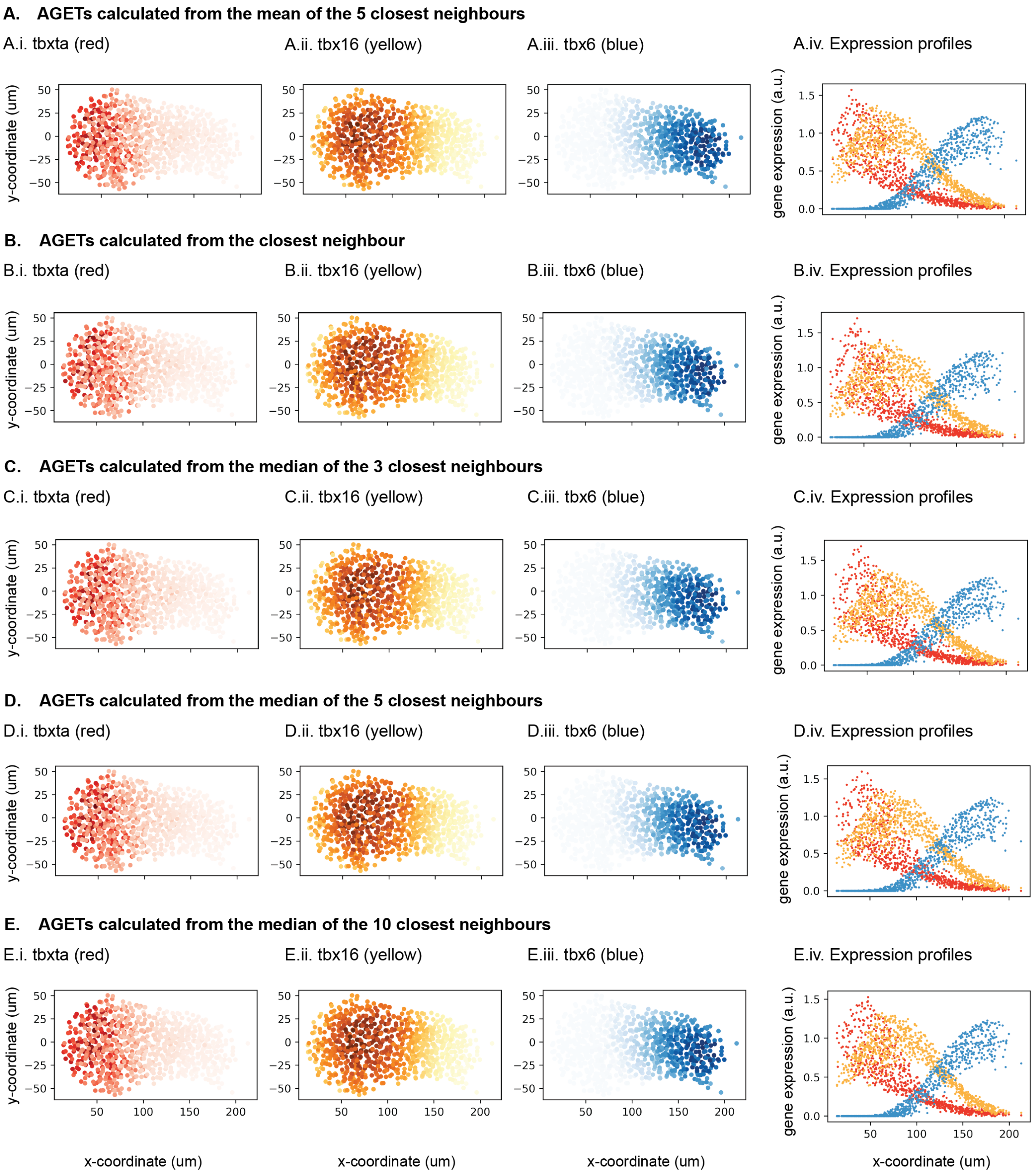

Figure S 3: **Tissue-level pattern is robust to AGET calculation method.** Related to Figure 3. **A.**Approximated Tbox gene expression pattern on the PSM when AGETs were calculated taking the mean of the five nearest neighbours. **A.i.** Approximated *tbxta* in the cells of the PSM. Each dot represents the position of a cell in the PSM (x,y-projection where dorsal is to the top and posterior is to the left). Shade of red indicates approximated *tbxta* concentration (dark red, highest, white, lowest). **A.ii.** Approximated *tbx16* in the cells of the PSM. Shade of yellow indicates approximated *tbx16* concentration (dark yellow, highest, white, lowest). **A.iii.** Approximated *tbx6* in the cells of the PSM. Shade of blue indicates approximated *tbx16* concentration (dark blue, highest, white, lowest). *Caption continues on the next page.*

Figure S 3: *Caption continues from the previous page.* **A.iv.** Tbox gene expression profiles. Each dot represents the concentration of one of the tbox genes (*tbxta* (red), *tbx16* (yellow) and *tbx6* (blue) in a given cell. The position along the posterior to anterior axis of each cell is given by its x-coordinate. **B.** Approximated Tbox gene expression pattern on the PSM when AGETs were calculated taking the value of the nearest neighbour.**B.i.** Approximated *tbxta* in the cells of the PSM. **B.ii.** Approximated *tbx16* in the cells of the PSM. **B.iii.** Approximated *tbx6* in the cells of the PSM. **B.iv.** Tbox gene expression profiles. **C.** Approximated Tbox gene expression pattern on the PSM when AGETs were calculated taking the value of the nearest neighbour.**C.i.** Approximated *tbxta* in the cells of the PSM. **C.ii.** Approximated *tbx16* in the cells of the PSM. **C.iii.** Approximated *tbx6* in the cells of the PSM. **C.iv.** Tbox gene expression profiles. **D.** Approximated Tbox gene expression pattern on the PSM when AGETs were calculated taking the value of the nearest neighbour.**D.i.** Approximated *tbxta* in the cells of the PSM. **D.ii.** Approximated *tbx16* in the cells of the PSM. **D.iii.** Approximated *tbx6* in the cells of the PSM. **D.iv.** Tbox gene expression profiles. **E.** Approximated Tbox gene expression pattern on the PSM when AGETs were calculated taking the value of the nearest neighbour.**E.i.** Approximated *tbxta* in the cells of the PSM. **E.ii.** Approximated *tbx16* in the cells of the PSM. **E.iii.** Approximated *tbx6* in the cells of the PSM. **E.iv.** Tbox gene expression profiles.

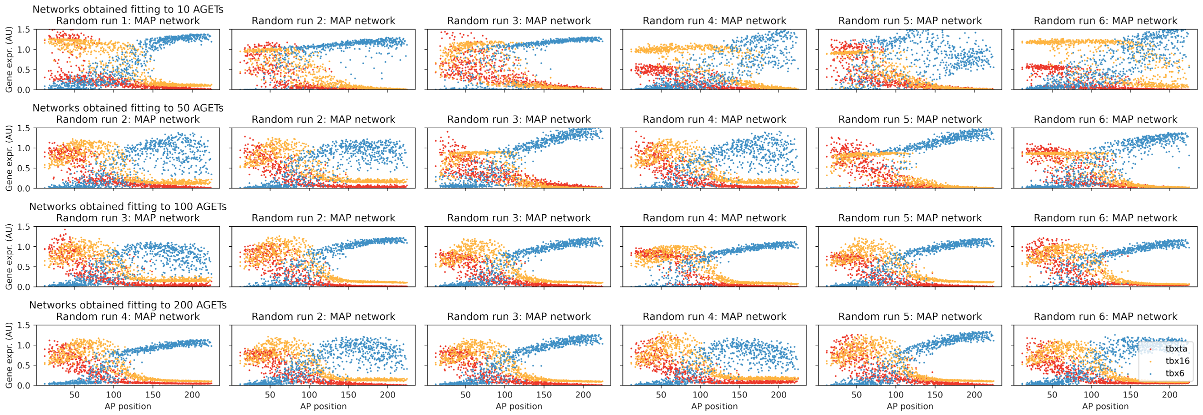

Figure S 4: **The proportion of parameter combinations producing good fits increases as the number of AGETs used for fitting is increased.** Related to Figure 3.**(A)** Networks obtained fitting to 10 AGETs. **(B)** Networks obtained fitting to 50 AGETs. **(C)** Networks obtained fitting to 100 AGETs. **(D)** Networks obtained fitting to 200 AGETs. In each case, the MAP parameter set is taken from 6 independent random runs and the expression profile corresponding to the last time point in the simulation is plotted. Each dot represents the concentration of one of the tbox genes (*tbxta* (red), *tbx16* (yellow) and *tbx6* (blue) in a given cell. The position along the posterior to anterior axis of each cell is given by its x-coordinate. Acceptable fits are obtained regardless of the number of AGETs used for fitting, but the proportion of acceptable fits increases with the number of AGETs.

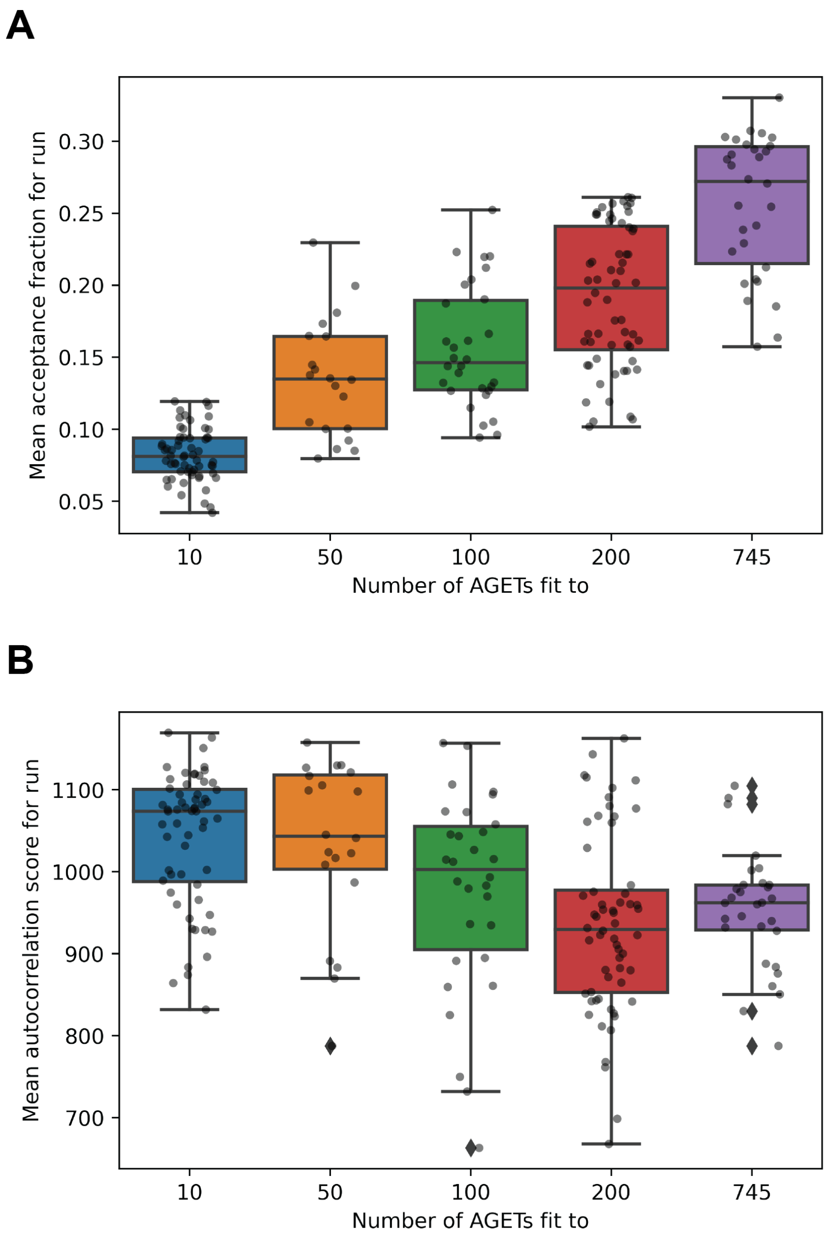

Figure S 5: **Fitting efficiency increases with the number of AGETs.** Related to Figure 3. **A.** Mean acceptance fraction per run increases with the number of AGETs used for fitting in initial tests. **B.** Mean auto-correlation score per run decreases as the number of AGETs increases until 200 AGETs, and stabilises thereafter.

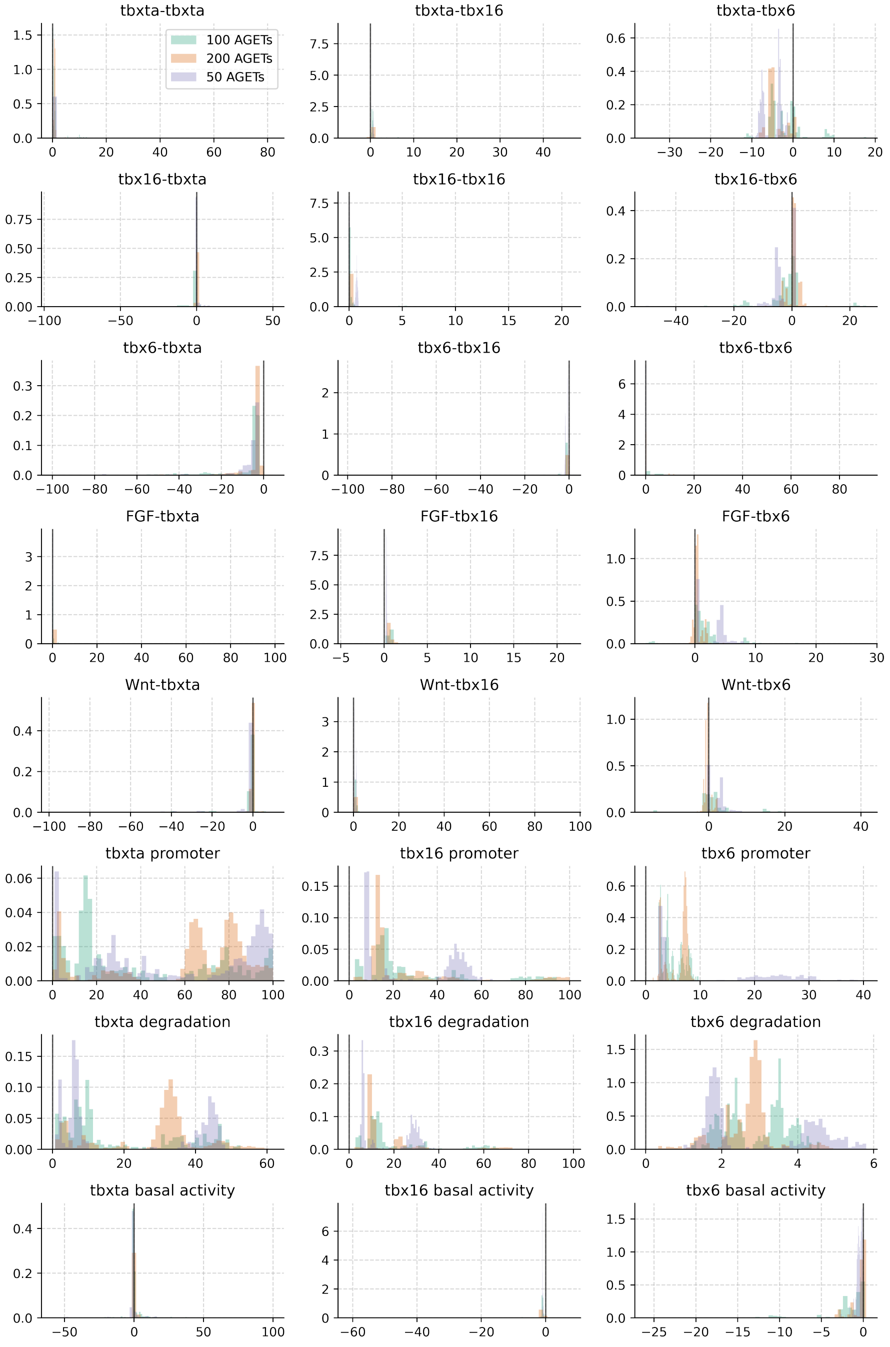

Figure S 6: **Marginal posterior distribution parameter values obtained using 10, 50, 100 and 200 AGETs.** Related to Figure 3.

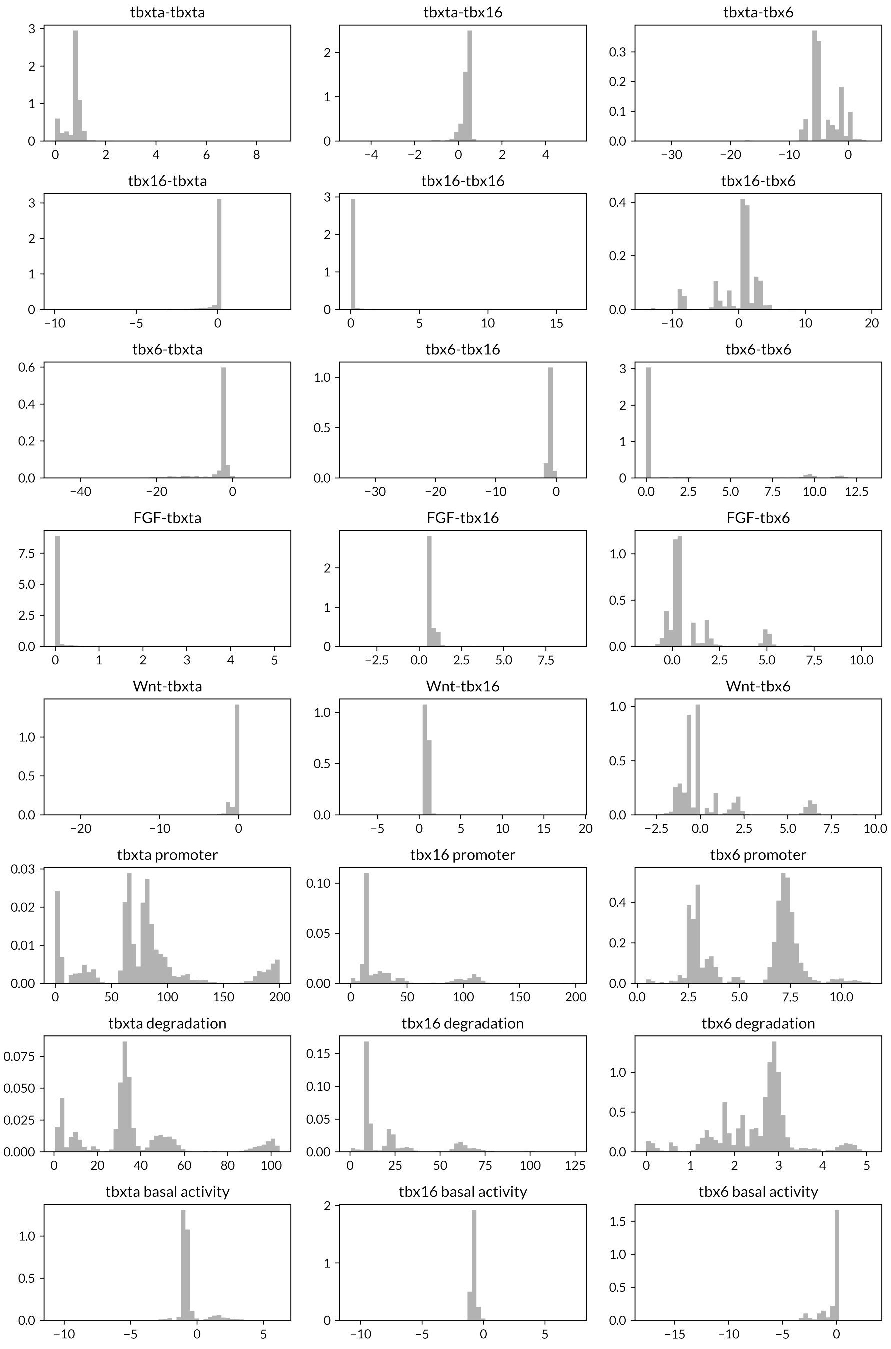

Figure S 7: **Marginal posterior distribution of filtered parameter values used for analysis and clustering.** Related to Figure 3.

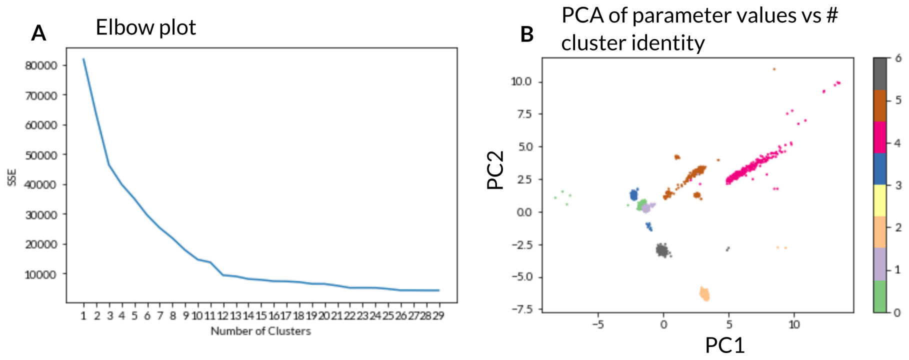

Figure S 8: **GRN Clustering.** Related to Figure 4. **A**. Elbow plot of SSE vs cluster size. **B.** PCA of parameter values, colour coded by cluster.
